# Ultrasonic-Based Quantification and Process Parameter Optimization of Anisotropy and Heterogeneity in WAAM 2319 Aluminum Alloy

**DOI:** 10.3390/ma19071433

**Published:** 2026-04-03

**Authors:** Chao Li, Hanlei Liu, Xinyan Wang, Jingjing He, Xuefei Guan

**Affiliations:** 1 AECC Huanan Aviation Powerplant Research Institute, Zhuzhou 412002, China; lc21th0124@163.com; 2 Graduate School of China Academy of Engineering Physics, Beijing 100191, China; liuhanlei22@gscaep.ac.cn; 3 AVIC Manufacturing Technology Institute, Beijing 100024, China; wangxinyan20@gscaep.ac.cn; 4 School of Reliability and Systems Engineering, Beihang University, Beijing 100193, China; hejingjing@buaa.edu.cn

**Keywords:** wire and arc additive manufacturing, ultrasonic evaluation, anisotropy, heterogeneity, process parameter optimization

## Abstract

Wire and arc additive manufacturing (WAAM) offers high deposition efficiency for large-scale aluminum components; however, layer-by-layer thermal cycling often induces microstructural anisotropy and spatial heterogeneity, which compromise structural reliability. In this study, an ultrasonic-based quantitative framework is proposed to evaluate and optimize anisotropy and heterogeneity in WAAM 2319 aluminum alloy. Nine blocks were fabricated using an orthogonal design with three key process parameters: torch travel speed, arc current, and shielding gas flow rate. Ultrasonic velocity and attenuation were employed to construct anisotropy and heterogeneity indicators. Results show that velocity-based anisotropy remains below 0.53%, indicating nearly isotropic elastic stiffness, whereas attenuation-based anisotropy reaches up to 76%, revealing pronounced direction-dependent microstructural and porosity features. Metallographic analysis confirms that grain morphology variation and interlayer porosity jointly govern attenuation responses. Response surface surrogate models were established to correlate ultrasonic indicators with process parameters, and both single- and multi-objective optimizations were performed within the feasible process window. The proposed framework provides a non-destructive, volumetric approach for microstructure-informed process parameter optimization in WAAM aluminum alloys.

## 1. Introduction

Metal additive manufacturing (AM) is increasingly adopted for rapid fabrication, component repair, and remanufacturing owing to its cost effectiveness and manufacturing flexibility [1]. Directed energy deposition (DED) is one of the major process families in metal AM. Wire and arc additive manufacturing (WAAM) uses an electric arc as the heat source and a metal wire as the feedstock, producing near-net-shape components by depositing material layer by layer on a substrate. Compared with laser- and electron-beam-based DED processes, WAAM typically provides higher deposition rates, lower feedstock cost, and reduced system complexity, which is advantageous for efficient manufacturing and repair of large-scale structures [2,3]. WAAM-processed aluminum alloys have received sustained attention because of their high specific strength and corrosion resistance. Among these alloys, 2319 (Al–Cu6.3) aluminum welding wire is widely used for WAAM owing to its demonstrated weldability and established engineering applications [4].

Under repeated thermal cycles associated with layer-by-layer deposition, WAAM aluminum alloys tend to develop microstructural heterogeneity and spatially nonuniform porosity, leading to property anisotropy and within-part variability in quality [5]. Anisotropy denotes the differences in microstructure manifested along different directions, while heterogeneity denotes the non-uniform spatial distribution of the microstructure within the same component. At the microstructural scale, thermal-history-driven changes in grain morphology and crystallographic texture result in direction- and location-dependent elastic and plastic responses [6]. At the defect scale, the large disparity in hydrogen solubility between liquid and solid states makes pore formation during solidification strongly process dependent and difficult to fully eliminate [7]. From an ultrasonic inspection standpoint, the microstructural and defect-related factors influence the sound velocity and anisotropy of the material while altering ultrasonic attenuation and scattering. Specifically, microstructural gradients and orientation-dependent elastic properties cause propagation behavior to vary with build direction and height, manifested by spatial variations in echo arrival time and amplitude [8,9,10,11,12,13]. In addition, porosity, a typical volumetric defect, can markedly increase scattering and energy dissipation, thereby reducing ultrasonic signal amplitude [14]. Accordingly, for WAAM 2319 aluminum, ultrasonic descriptors and an inversion framework are required to couple microstructural anisotropy with porosity characteristics and to support evaluation of quality consistency and in-service reliability [15,16,17,18,19,20].

Recent studies indicate that mechanical anisotropy and heterogeneity are prevalent in AM components and are commonly attributed to the coupled effects of directionally dependent microstructural features and internal defects. Hossain et al. [21] fabricated nominally isotropic stochastic porous structures in stainless steel (SS316L) and titanium alloy (Ti-6Al-4V) via laser powder bed fusion, and measured elastic modulus and yield strength along 10 orientations for each material. Despite the intended isotropy, pronounced anisotropy was observed; the standard deviation across the 10 measurements was used to quantify anisotropy magnitude. Similar orientation-dependent behavior has also been reported in arc-based AM processes. Zhang et al. [22] produced Al-6Mg alloy blocks using variable-polarity cold metal transfer (VP-CMT) under different arc modes and reported tensile strength anisotropy of approximately 8–27% across specimen orientations. This anisotropy was primarily attributed to interlayer microporosity. Chen et al. [23] examined the tensile behavior of DED-fabricated 316L stainless steel in two orientations (parallel and perpendicular to the build direction) and observed anisotropic tensile responses linked to distinct grain morphologies arising from different solidification paths during AM. Arana et al. [24] proposed process strategies to reduce porosity in Al–Mg WAAM builds and assessed associated mechanical implications. Direction-dependent behavior was observed, with ultimate tensile strength anisotropy below 9%, whereas elongation anisotropy reached up to 11% at higher porosity levels, indicating a key role of porosity in WAAM-induced anisotropy. For a WAAM-fabricated Hastelloy C276 thin-wall structure, Qiu et al. [25] assessed microstructure and mechanical properties using specimens extracted from different orientations and locations. Results showed that microstructural variations along the build direction drive anisotropy and nonuniformity in mechanical performance. In parallel, NDE methods have been explored to quantify such spatial nonuniformity and anisotropy in WAAM components. Liu et al. [26] used phased-array ultrasonic backscatter to characterize spatial variation and directionality in a WAAM 2319 aluminum alloy. Multi-angle backscatter datasets were acquired via electronic focusing and beam steering; path-integrated backscatter amplitude and the root-mean-square of directional backscatter were used to quantify microstructural nonuniformity and anisotropy, respectively. The WAAM 2319 sample exhibited nonuniformity with weak anisotropy, and complementary metallography, EBSD, and XCT indicated that the backscatter response is jointly governed by grains/texture and porosity, with non-negligible porosity contributions. Lopez et al. [27] applied radiographic and ultrasonic testing to a quality assessment of WAAM components, demonstrating that both modalities can detect typical internal defects produced during WAAM, thereby supporting the use of NDE signals for process–quality control.

In the above research, microstructural anisotropy and heterogeneity are commonly assessed through localized, destructive methods such as metallography and mechanical testing. These conventional approaches, compared with non-destructive methods, have limited spatial coverage and statistical representativeness. For large-scale components, repeated sampling and testing at multiple locations are often required, resulting in high cost and long lead times. Moreover, these methods provide limited continuous information, which impedes reliable modeling of process–quality relationships and inverse optimization of process parameters. These limitations motivate volumetric, non-destructive alternatives for WAAM characterization. In contrast, ultrasonic NDE is non-destructive and high throughput, providing rich inspection data for rapid evaluation of WAAM components. Prior studies have shown that phased-array ultrasonics and related imaging techniques can detect and characterize defects in WAAM aluminum alloys and can support interpretable links between ultrasonic responses and microstructural and porosity features [17,26,27,28,29,30]. Building on these advances, this study develops an ultrasonic NDE-based characterization framework to enable process parameter optimization. Phased-array ultrasonics is used to extract features and construct ultrasonic metrics that quantify anisotropy and heterogeneity; a surrogate model is then developed to map process parameters to these metrics. Single- and multi-objective optimization are subsequently performed within the feasible process window to identify parameter sets optimal under different design preferences.

This paper proposes a systematic methodology for the process parameter optimization of WAAM 2319 aluminum alloy. Phased-array ultrasonics is employed to provide quantitative evaluation of microstructural anisotropy and heterogeneity along two orthogonal principal directions; optimal parameter combinations are then identified within the feasible process window. The remainder of this paper is organized as follows. First, nine design points are defined based on three key process parameters, and WAAM bulk specimens are fabricated accordingly. Phased-array ultrasonic inspections are then performed to acquire datasets, from which sound velocity and attenuation coefficients are calculated; these quantities are used to develop quantitative ultrasonic metrics and an evaluation workflow for anisotropy and heterogeneity. In parallel, metallographic characterization is conducted to elucidate the combined influence of spatially nonuniform porosity and microstructural variations on the proposed ultrasonic metrics. A response surface-based surrogate model is then established to describe the relationship between process parameters and ultrasonic metrics, and both single- and multi-objective optimizations are conducted to guide the selection of process parameters, such as torch travel speed, arc current, and shielding gas flow rate, for different design objectives. Finally, conclusions are drawn from the current results.

## 2. Material Preparation

In this study, the cold metal transfer with the addition of pulses (CMT+P)-based WAAM is chosen for the fabrication of aluminum alloy components. The performance of the components manufactured by CMT+P-based WAAM depends on the process control parameters. In practical engineering applications, three process parameters, namely, the arc current, the torch travel speed, and the shielding gas (99.99–pure argon) flow rate, are adjustable in their valid ranges. Therefore, these three parameters are considered as main influencing variables in the WAAM process. To investigate the impact of the three parameters on the anisotropy and heterogeneity microstructure of as-built material, a total number of nine blocks were fabricated using different combinations of the three parameters.

### 2.1. Material and Specimen Fabrication

**Figure 1:**
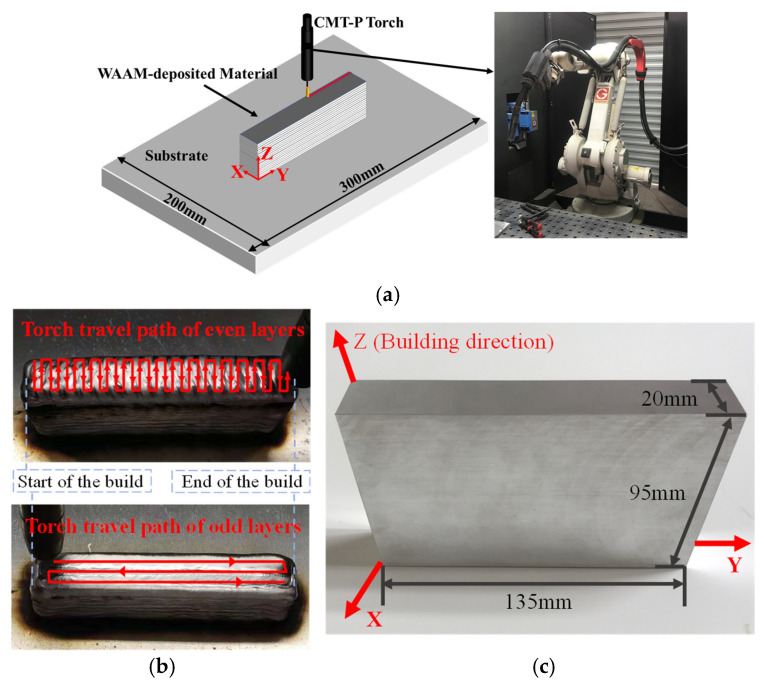
(**a**) Schematic and actual setup of the WAAM system, (**b**) oscillation strategy for torch travelling path, and (**c**) one sample of the built block.

The in-house built manufacturing system for the fabrication of WAAM blocks is shown in Figure 1a. The CMT+P arc is employed to deposit ER2319 aluminum wires with a diameter of 1.2 mm. The chemical composition of the wire is shown in Table 1.

A torch that feeds the wire and supplies the shielding gas simultaneously is driven by a six-axis robot arm to build the material blocks layer by layer. A 2219-T87 aluminum alloy plate with dimensions of 300 mm × 200 mm × 20 mm is used as the substrate. To reduce the effect of the path dependence of the final part, an oscillation strategy is adopted for the torch traveling path in each of the layers. In addition, the main oscillation directions for any two adjacent layers are kept orthogonal, as illustrated in Figure 1b. The WAAM blocks are fabricated using each of the nine design points for the three control parameters and are sectioned by wire electrical discharge machining (WEDM) to obtain clear surfaces for characterization. The final dimensions of all specimens are the same as shown in Figure 1c, where the building direction is defined as Z-axis, and X-axis and Y-axis are the two coordinates within the deposited layers.

### 2.2. Selection of Process Parameters

**Figure 2:**
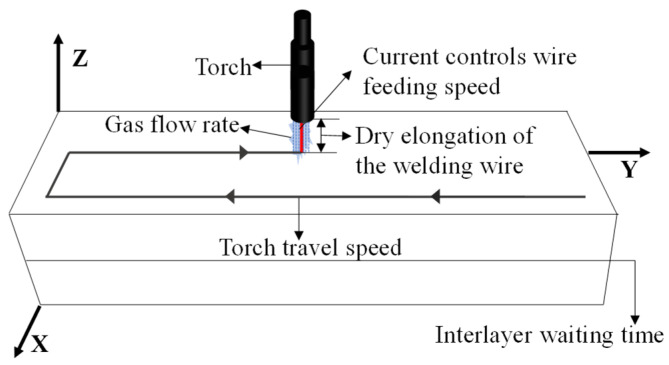
Schematic diagram of the WAAM process showing the main process parameters.

**Figure 3:**
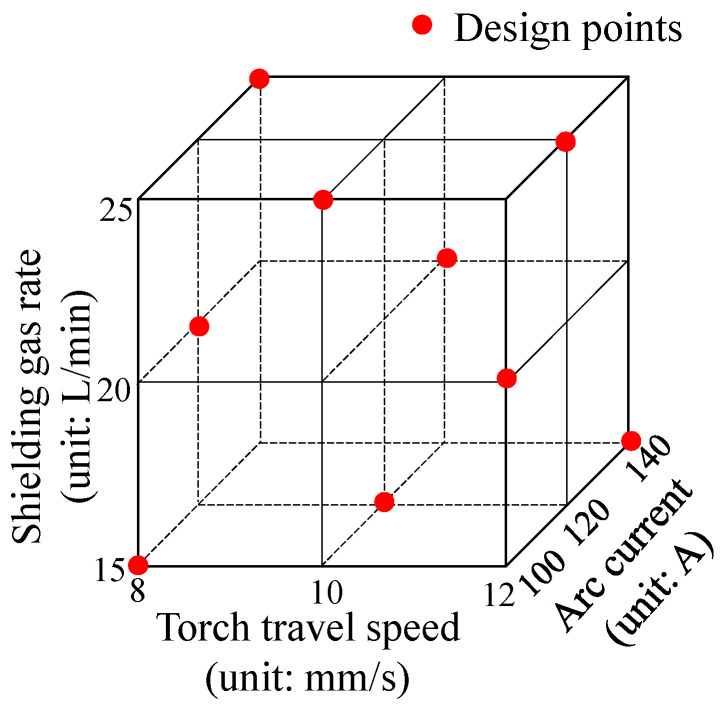
Design points in the joint parameter space.

The main WAAM process parameters, including arc current, wire diameter, wire feed rate, torch travel speed, and shielding gas flow rate, are schematically illustrated in Figure 2. According to Gu et al. [15] and Fang et al. [31], three process parameters, namely torch travel speed labeled v, arc current labeled I, and shielding gas flow rate labeled Q, are important factors affecting the performance of the fabricated part. To investigate the effects of these three parameters on microstructural anisotropy and heterogeneity, an L_9_ orthogonal array with three factors at three levels is adopted, as shown in Table 2. The nine design points in the joint parameter space are shown in Figure 3. The parameter ranges from Level 1 to Level 3 are determined based on hardware constraints and engineering experience.

**Figure 4:**
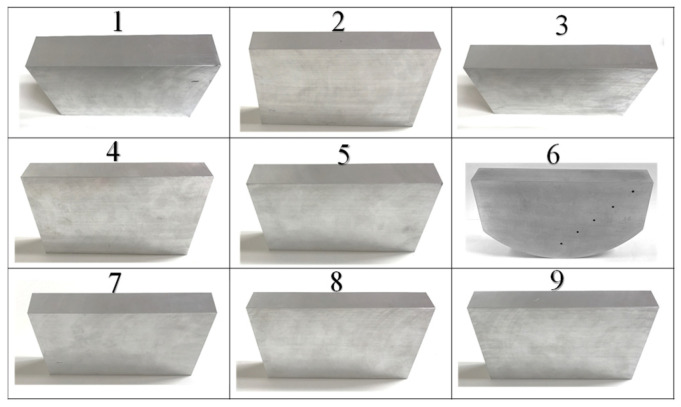
WAAM blocks fabricated according to the process parameters in Table 3.

A total of nine WAAM blocks were fabricated according to the nine design points of the parameters. The actual recorded values of process parameters associated with the fabricated specimens are shown in Table 3. It is noted that the actual arc current values are slightly different from the design values of the three levels due to the precision limit of the hardware. Other minor process parameters are kept constant. For example, the dry elongation of the welding wire is 12 mm and the wait time between subsequent layer depositions is 30 s. Therefore, the difference in the anisotropy and heterogeneity in the microstructures of the resulting nine blocks can be entirely attributed to the combination of the three process parameters. The fabricated material blocks were further machined to form clean surfaces and are shown in Figure 4. The block No. 6 is further machined to have a round surface for the sake of the calibration of the phased-array system. Therefore, the velocity and attenuation measurements are only accessible via the side surface for this block.

## 3. Ultrasonic-Based Quantification for Anisotropy and Heterogeneity

The ultrasonic velocity and attenuation-based indicators are proposed to quantify the anisotropy and heterogeneity of the material. Phased-array ultrasonic testing is performed on the WAAM blocks, and the ultrasonic data along two orthogonal principal directions (along Y/Z-axis) are obtained. Metallography is subsequently performed on one material block along the two major directions. Microstructural features, including pores and grain morphology, are characterized to validate the effectiveness of the ultrasonic based indicators.

### 3.1. Theoretical and Empirical Relations

Quintana et al. [32] reported that the ultrasonic velocity V is related to the elastic modulus component and the material density, as follows:

(1) $$V=\sqrt{\frac{C_{11}}{\rho }}$$

where the terms $C_{11}$ is the elasticity moduli component in the wave/loading direction and ρ is the material density. In addition, Nanekar et al. [33] established the correspondence between the attenuation coefficient α and the microstructure features of the material such as grain size via

(2) $$\alpha =C_{r}\cdot D^{3}\cdot f^{4},$$

where $C_{r}$ is the scattering parameter depending on the type of wave used, D is the grain size on average, and f is the frequency. The selection of $C_{11}$ and $C_{r}$ is based on different characteristics. $C_{11}\;$ reflects direction-dependent stiffness variation associated with anisotropy, whereas $C_{r}$ reflects scattering-dominated attenuation behavior associated with heterogeneity. Slotwinski et al. [34] reported that for material having non-negligible porosity φ (in volume percent), the velocity V can be empirically modified using the following equation:

(3) $$V=v_{0}+\beta \varphi ,$$

where $v_{0}$ is the ultrasonic velocity for a no-void sample, and β is the slope of the linear fit. Lin et al. [35] established the attenuation coefficient α in case of having non-negligible porosity as

(4) $$\begin{array}{c} \alpha =\left\{\begin{array}{c} a+b\varphi ,\;\;\;\;\;\;\;\;\;\;\;\;\;\;\;\;\;\;\;\;\;\;\;\;\;\;0.03\%\leq \varphi \leq 0.5\%\\ c_{1}+c_{2}\varphi +c_{3}\varphi^{2},\;\;\;\;\;\;\;\;\;\;0.5\%\leq \varphi \leq 4.96\% \end{array}\right. \end{array}$$

where the terms $a,b,c_{1},\;c_{2},\;c_{3}$ are determined by experimental data on a fully dense sample. The above relations provide physical support for using ultrasonic velocity and attenuation as complementary indicators of anisotropy and heterogeneity in the material, respectively, while the attenuation–porosity relation is cited only as an empirical reference.

**Figure 5:**
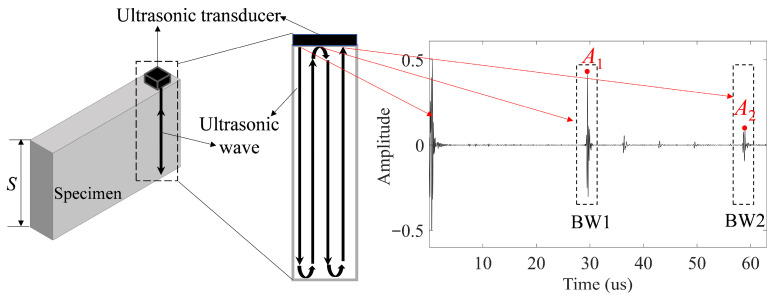
Schematic description of the ultrasonic pulse-echo method, and a representative time-domain signal showing the first and second back-wall echoes.

In this study, the ultrasonic pulse-echo method is adopted to acquire ultrasonic data, as shown in Figure 5. The ultrasound generated by a transducer propagates through a specimen with a thickness of *S* and reflects as it encounters the back wall of the specimen. In this case, the first back-wall echo (BW1) with a traveled distance of 2*S*, and the second back-wall echo (BW2) with a traveled distance of 4*S*, are acquired by the transducer. A sample of the acquired data is shown in Figure 5. The one-dimensional signal is also referred to as A-scan in the context of ultrasound non-destructive evaluation. The ultrasound wave velocity (V) can be calculated by the time of flight (TOF) between two back-wall echoes and the traveled distance of 2*S* as, according to the standard ASTM E494 [36],

(5) $$V=\frac{2S}{TOF}.$$

When the ultrasound propagates in the specimen, its total energy dissipates due to the natural energy losses and the scattering at the weak microstructural discontinuities, such as grain boundaries, the interface of two phases, pores, and so on. The actual attenuation coefficient, denoted as α, can be used to quantify the total loss of energy owing to the absorption and scattering along the propagation path:

(6) $$\alpha =\frac{1}{2S}\ln\frac{A_{1}}{A_{2}},$$

where the term *S* is the thickness along the wave propagation direction of the specimen, $A_{1}$ is the amplitude of the BW1, and $A_{2}$ is the amplitude of the BW2, as illustrated in Figure 5.

#### 3.1.1. Anisotropy Indicator

Anisotropy describes differences in orientation-dependent material features, as emphasized by Kok et al. [9]. Javidrad et al. [37] reported that direction-dependent microstructural features can arise from the path-dependent layer-by-layer deposition process in WAAM.

**Figure 6:**
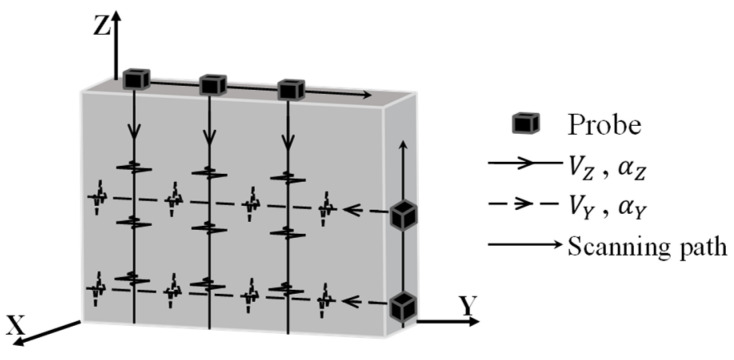
Schematic diagram of ultrasound propagating along two orthogonal directions.

Two orthogonal directions, parallel/perpendicular to the building direction Z-/Y-axis shown in Figure 1, are considered as the typical orientations to characterize the anisotropy in the microstructure. Ultrasonic testing is performed on the fabricated block to obtain the velocity $V_{Z}$ and attenuation coefficient $\alpha_{Z}$ of the ultrasound propagating along the building direction, as shown in Figure 6. The volumetric testing is achieved by continuously acquiring the data at fixed step length along the scanning path of the transducer. At each sampling location, a specific velocity and attenuation coefficient are obtained, and the mean value ${\bar{V}}_{Z}$ and ${\bar{\alpha }}_{Z}$ can be computed using data at all sampling locations. In the same fashion, the mean value of velocity ${\bar{V}}_{Y}$ and mean value of attenuation coefficient ${\bar{\alpha }}_{Y}$ of the ultrasound wave propagating parallel to the Y-axis can also be determined.

The anisotropy indicators based on the velocity and the attenuation coefficient are proposed as

(7) $$A_{V}=\frac{\left|{\bar{V}}_{Z}-{\bar{V}}_{Y}\right|}{\left({\bar{V}}_{Z}+{\bar{V}}_{Y}\right)/2},$$

and

(8) $$A_{\alpha }=\frac{\left|{\bar{\alpha }}_{Z}-{\bar{\alpha }}_{Y}\right|}{\left({\bar{\alpha }}_{Z}+{\bar{\alpha }}_{Y}\right)/2},$$

respectively. In the above two equations, the terms $\left|{\bar{V}}_{Z}-{\bar{V}}_{Y}\right|$ and $\left|{\bar{\alpha }}_{Z}-{\bar{\alpha }}_{Y}\right|$ measure the difference in the velocity and attenuation coefficient of the ultrasound wave propagated along two orthogonal directions, respectively. The terms $\left({\bar{V}}_{Y}+{\bar{V}}_{Y}\right)/2$ and $\left({\bar{\alpha }}_{Z}+{\bar{\alpha }}_{Y}\right)/2$ loosely measure the average velocity and the average attenuation coefficient of the material, respectively.

#### 3.1.2. Heterogeneity Indicator

The assumption that a material is homogeneous in its properties is widely used to simplify the design and assessment of engineering components. For conventionally manufactured materials, this assumption is usually valid. However, Wang et al. [38] reported that the location dependence of material properties in WAAM parts cannot be neglected. The degree of heterogeneity can be quantified by modeling spatial variations in material properties. In this study, property variations along the Z and Y directions of WAAM components are used to quantify heterogeneity for method development. This procedure can be applied in any selected direction.

As shown in Figure 6, the ultrasonic system acquires the data sequentially along the scanning path parallel to the Z-axis and Y-axis. The velocity and attenuation coefficient are extracted using the data acquired at the sampling location at each of the sampling locations along the path. The mean value (MV) and the standard deviation (SD) of the velocity and attenuation coefficient can subsequently be calculated using results at all sampling locations. SD reflects the degree of deviation of the samples from the mean value MV. To compare the degree of heterogeneity among different material blocks, the coefficient of variation CV is adopted as a statistical indicator of heterogeneity, which is defined as

(9) $$CV(x)=\frac{SD(x)}{MV(x)},$$

where x is the variable of interest. The heterogeneity in this case can be quantified using the coefficient of variation of velocity CV(V) and the coefficient of variation of attenuation coefficient CV(α).

### 3.2. Phased-Array Ultrasonic Testing

**Figure 7:**
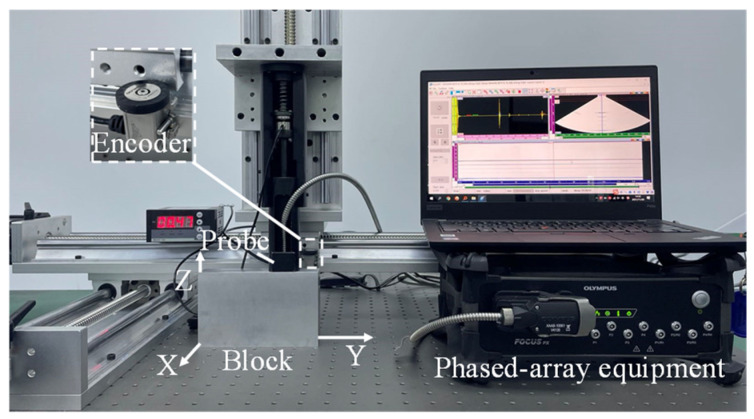
The ultrasonic testing setup.

Phased-array ultrasonic testing is performed on the WAAM 2319 aluminum alloy blocks to obtain ultrasonic data. The phased-array ultrasonic testing setup is shown in Figure 7. An Olympus Focus PX phased-array system (Evident, Tokyo, Japan) equipped with a 16-element phased-array transducer with a central frequency of 5 MHz and a linear position encoder is used for pulse-echo mode data acquisition. The phased-array probe is coupled to the block surface using a liquid couplant to ensure reliable contact during testing. Ultrasonic beams at 0 degrees, perpendicular to the inspection surface, are generated by the phased-array controller at a sampling frequency of 100 MHz. To acquire data in the Z-axis direction, the probe is moved along the center line of the inspection surface X–Y in a three-axis platform. A linear position encoder triggers the probe to transmit ultrasound into the material and acquire the resulting data whenever the probe is moved a prescribed step size. In this study, the step size along the scanning path is 0.247 mm. Therefore, the material is densely sampled along the scanning path. The same testing process is performed to acquire data in the Y-axis direction by moving the probe along the center line of the inspection surface X–Z.

**Figure 8:**
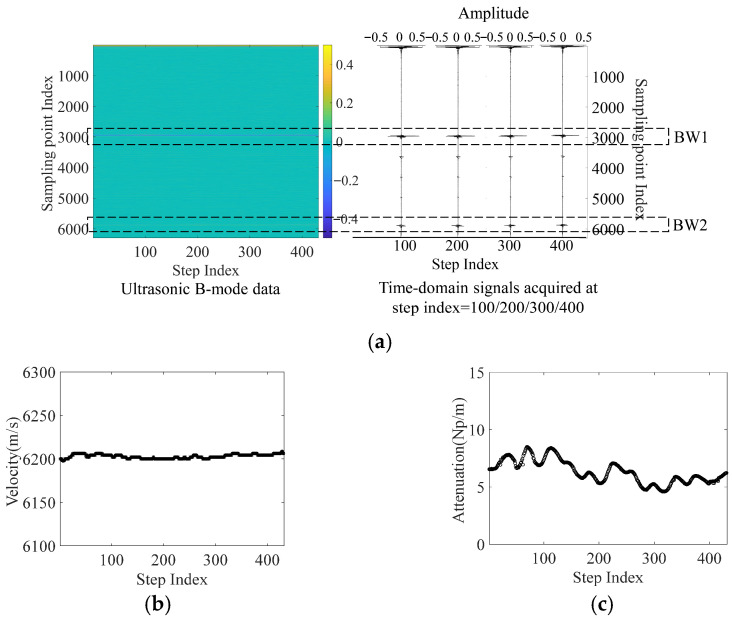
(**a**) Ultrasonic inspection data of block No. 9 along the scanning path parallel to the Y-axis and the A-scan data at steps indexed at 100/200/300/400, (**b**) velocity variation along the scanning path, and (**c**) attenuation coefficient variation along the scanning path.

Ultrasonic inspections are performed along the two orthogonal orientations (Z/Y-axis) for each of the nine blocks. For illustration, the entire ultrasonic data acquired on the inspection surface X–Y of block No. 9 is shown in Figure 8a as an intensity image. In the image, the horizontal direction is the scanning direction, and the vertical direction is the direction of the beam axis. Each vertical line is a one-dimensional A-scan acquired at a sampling location along the scanning path. The acquired A-scan data are arranged in the sampling order to form a two-dimensional matrix. The two-dimensional matrix is visualized as an intensity image by mapping the intensity, namely the echo amplitude, to a colormap shown on the color bar. For the A-scan data at each of the sampling positions along the scanning path, the BW1 and BW2 in the time domain are extracted by choosing the appropriate time gates, and their peak values are identified as the amplitude $A_{1}$ and $A_{2}$. The amplitude values of the two peaks and the TOF between them are used to calculate the velocity and attenuation coefficient according to Equation (5) and Equation (6), respectively. The calculated velocity and attenuation coefficient using the data in Figure 8a are shown in Figure 8b and Figure 8c, respectively. The mean value and standard deviation of the velocity are 6202.9 m/s and 2.3693, respectively. The mean value and standard deviation of the attenuation coefficient are 6.3263 Np/m and 1.0032, respectively.

### 3.3. Anisotropy and Heterogeneity Evaluation of WAAM 2319 Aluminum Alloy

The velocity (V) and attenuation coefficients (α) of the two orthogonal directions for each of the nine blocks are evaluated using Equation (5) and Equation (6), respectively. The mean and standard deviation of V and α on each of the scanning paths, and the corresponding coefficient of variation, are calculated using Equation (9).

The results for ultrasound velocity are shown in Table 4. The results associated with the Z-axis direction of block No. 6 are not available for the reason described in Section 2.2. The terms ${CV(V}_{Z})$ and ${CV(V}_{Y})$ are the coefficients of variation of velocity on the scanning paths perpendicular to the Z-axis and Y-axis, respectively. The velocity-based anisotropy values, $A_{V}$, are all below 0.53%, indicating that the WAAM 2319 aluminum alloy exhibits nearly isotropic elastic stiffness along the two orthogonal directions according to the anisotropy criteria used in ISO 19675 [39]. The values of the coefficient of variation ${CV(V}_{Z})$ and ${CV(V}_{Y})$ are less than 0.82% in the two directions, implying a small scatter of velocity data around the mean along the scanning paths. Based on the criteria [40], the material is considered as homogeneous for a CV value less than 3%. By using the theoretical relationship of Equation (1), it is reasonable to conclude that these WAAM blocks are homogeneous in elastic modulus along the Z-axis and Y-axis. However, the criterion for heterogeneity is not universal across all materials and depends on the design requirements.

The back and forth oscillation within a layer and the orthogonal oscillation between adjacent layers are believed to contribute substantially to overall uniformity [41]. In particular, the ${CV(V}_{Z})\;$ results of the nine blocks are less than 0.4%. The slightly heterogeneous microstructure caused by different cyclic thermal histories along the building direction contributes to a higher ${CV(V}_{Y})$ [9].

The attenuation-based results are presented in Table 5. The terms $CV(\alpha_{Z})$ and $CV(\alpha_{Y})$ are the coefficients of variation of the attenuation coefficient associated with the scanning path perpendicular to the Z-axis and Y-axis, respectively. The values of the attenuation-coefficient-related parameters of different blocks are vastly different. As the attenuation of the ultrasound is mainly affected by the microstructural features of the materials, such as the grain boundaries, inclusions, phase interfaces, porosities, and so on, a large difference in the attenuation coefficient among the specimens indicates that the attenuation coefficient is much more sensitive to the microstructural features than the velocity. A larger $A_{\alpha }$ implies a greater difference in the microstructural features of the two orthogonal directions. Notably, attenuation anisotropy does not directly imply elastic stiffness anisotropy, since attenuation is dominated by scattering from microstructural heterogeneities rather than bulk stiffness. Under the same torch travel speed, the increased arc current leads to the increase of the total heat input. The time for crystal grains to recrystallize becomes longer and promotes a larger grain size, leading to a greater difference in the microstructure along the two orthogonal directions.

It should be emphasized that the anisotropy indicators derived from ultrasonic velocity and attenuation reflect different physical mechanisms. Velocity is primarily governed by elastic stiffness and density, and therefore characterizes anisotropy in elastic modulus. In contrast, attenuation is strongly influenced by scattering and absorption caused by grain morphology, phase interfaces, and porosity. As a result, velocity-based anisotropy represents macroscopic elastic anisotropy, whereas attenuation-based anisotropy captures microstructural and defect-related anisotropy. In contrast to the velocity results, attenuation-based anisotropy reaches values as high as 76%, indicating strong direction-dependent microstructural and porosity features. This discrepancy suggests that although the macroscopic elastic response remains nearly isotropic, the underlying microstructure exhibits pronounced orientation-dependent heterogeneity. The degree of heterogeneity along the building direction, $CV(\alpha_{Y})$, is greater than that along the direction parallel to the Y-axis, $CV(\alpha_{Z})$. The same phenomenon has been reported in microstructural studies of the same material, including Cong et al. [42] and Gu et al. [15]. The changes in the grain morphology, such as size and shape, along with the building direction and the existence of pores, collectively affect the attenuation coefficient. The detailed quantitative metallographic analysis is presented next.

### 3.4. Metallographic Microstructure Characterization

**Figure 9:**
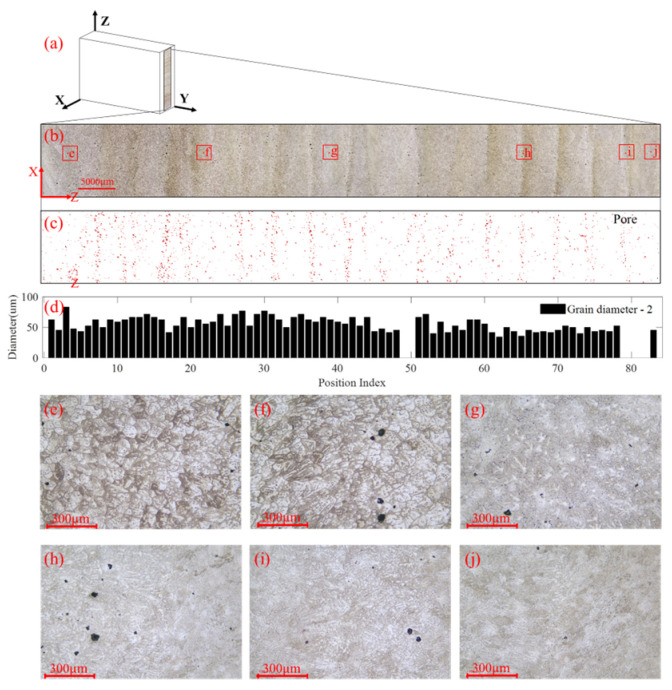
Microstructural features across layers of block No. 2. (**a**) Microscopic imaging on the X–Z cross-section, (**b**) the stitched whole surface using the microscopic images, (**c**) distribution of pores, (**d**) distribution of grain size, and (**e**–**j**) microstructures at boxed regions shown in (**b**).

**Figure 10:**
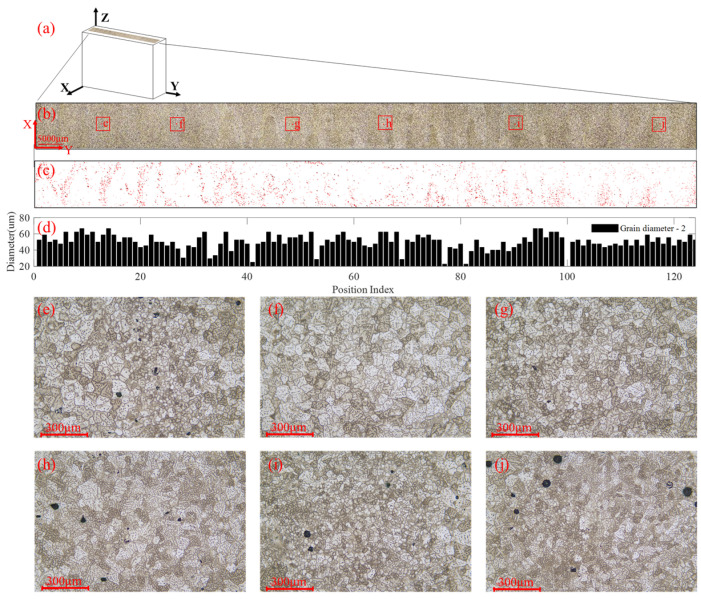
Microstructural features across layers of block No. 2. (**a**) Microscopic imaging on the X–Y cross-section, (**b**) the stitched whole surface using the microscopic images, (**c**) distribution of pores, (**d**) distribution of grain size, and (**e**–**j**) microstructures at boxed regions shown in (**b**).

To correlate the ultrasonic attenuation results with local microstructural features and potential manufacturing pores, metallographic analysis is performed. Block No. 2 is arbitrarily chosen for metallographic analysis. The metallographic specimens are sectioned from the deposited block along the X–Z and X–Y planes by wire electrical discharge machining. The samples are then hot mounted, mechanically ground and polished, etched using Keller’s reagent for 20 s, and finally cleaned with ethanol and dried. The optical microscopy imaging on the X–Z cross-section and X–Y cross-section is obtained using a Leica metallographic microscope, and the results are shown in Figure 9 and Figure 10, respectively. The imaging is made at an interval of 1 mm in both directions to ensure a sufficient resolution to capture the detail across the whole area. Porosity and grain size measurements are carried out within the two planes using the microscopic images and professional image analysis software. Spatial variations of pores and grain morphology can be analyzed.

#### 3.4.1. Interlayer Microstructural Features

The captured X–Z cross-section microstructure image is shown in Figure 9b. Pores in Figure 9b are identified and are shown in Figure 9c as red dots. The average diameter of the pores is 36 µm. For the X–Z cross-section of block No. 2, the average porosity is 0.2907%, and the coefficient of variation of porosity along the Z-axis is 0.9739. Pores are observed as banded clusters enriched at the boundaries of deposited layers, which are related to hydrogen evolution during solidification. This phenomenon is referred to here as a mechanism of pore formation, because the solubility of hydrogen in liquid aluminum is much higher than that in solid aluminum. Due to the large difference in the solubility of hydrogen in liquid aluminum and solid aluminum, the hydrogen is released when aluminum solidifies. Some hydrogen cannot escape in time and becomes trapped in the solid as bubbles, forming spherical pores [43], as shown in Figure 9f. A small number of irregularly shaped pores are shrinkage pores as shown in Figure 9g, which are caused by hydrogen migration into the voids caused by volume differences between the solid and liquid aluminum alloy [44].

The grain size along the building direction is measured using the intercept method in accordance with ASTM E112 [45], and the results are shown in Figure 9d. The average grain size is 55.6 µm, and the coefficient of variation of the grain diameter is 0.2021. The grain morphology varies significantly along the building direction. Specifically, equiaxed non-dendritic crystals, columnar crystals, dendrites, and columnar crystals with a large aspect ratio are observed from the bottom region to the top region, as shown in Figure 9e–j. In the bottom region close to the substrate, the crystals are mainly equiaxed because heat is rapidly dissipated through the substrate and the deposited material is further influenced by subsequent thermal cycling, as shown in Figure 9e. In the interlayer region shown in Figure 9f, a small number of columnar crystals are observed because of the relatively high temperature gradient in this area. With increasing deposition height, a thermal balance is gradually established, and equiaxed dendrites grow more freely without obvious preferential orientation, as shown in Figure 9g. Figure 9i exhibits a relatively dense dendritic morphology. In the top region, columnar crystals with a large aspect ratio are generated because of the strong temperature gradient, as shown in Figure 9j. Noticeable microstructural changes are also observed at the layer boundaries. For example, columnar crystals and equiaxed non-dendritic crystals coexist in Figure 9f, whereas equiaxed dendrites and equiaxed non-dendritic crystals coexist in Figure 9h. These observations indicate that, in the WAAM process, the previously deposited layers are repeatedly affected by the heat input from subsequent depositions, resulting in distinct microstructural features at different building heights. These microstructural observations help explain the statistical response characteristics discussed above. In particular, the non-uniform distribution of pores and the variation in grain morphology along the building direction contribute directly to the heterogeneity of the deposited material, while the directional evolution of the grain structures is closely related to the anisotropy response. Therefore, the microstructural features shown in Figure 9 provide physical support for the combined optimization of anisotropy and heterogeneity.

#### 3.4.2. Intralayer Microstructural Features

Figure 10b presents the overall X–Z cross-section microstructure image where pores are formed around the junction of two adjacent beads. The average pore diameter is 30 μm, and the average porosity is 0.2713% with a coefficient of variation of 0.6902 along the Y-axis. The grain morphology consists of equiaxed grains shown Figure 10f, cellular grains shown in Figure 10i, and equiaxed dendrites shown Figure 10h. The change in grain morphology shown in Figure 10e,g,j are caused by the thermal cycles exerted on the deposited material. Equiaxed grains are re-melted in the deposition process of subsequent beads. The grain size is refined, and small cellular grains are generated. The measured grain sizes along the Y-axis are shown Figure 10j. The average grain size is 50.44 μm, and the coefficient of variation of the grain diameter is 0.1812.

The above metallography results show that the porosity and grain morphology of the material varies greatly across the cross-section. The non-uniform spatial distribution of the pores and the variations of the microstructural features together cause the anisotropy and heterogeneity in the material. The measured metallographic results of the coefficient of variation of porosity and grain size in the X–Z cross-section are both greater than those in the X–Y section, which is consistent with the ultrasonic evaluation results, e.g., ${CV(\alpha }_{Y})>{CV(\alpha }_{Z})$. The change in the attenuation coefficient in the cross-section shown in Figure 8c is a result of the change in microstructural features shown in Figure 10. The significant variation in the attenuation coefficient shows that this ultrasonic parameter is highly sensitive to changes in microstructural features and therefore can be used as an effective indicator for the quantification of heterogeneity. To achieve optimal performance in terms of the anisotropy and heterogeneity of WAAM materials, models that can correlate the ultrasonic indicators with the WAAM process parameters are established and presented next.

## 4. WAAM Process Parameter Optimization

Three response surface surrogate models are established to correlate the ultrasonic indicators with the WAAM process parameters, allowing for inverse identification of the optimal parameters under different goals of optimization. Single- and multi-objective optimization are presented to demonstrate the overall method.

### 4.1. Surrogate Modeling Based on Response Surface Method

The response surface method (RSM) is adopted in this study to construct a mathematical model [46,47]. He et al. [48] noted that polynomial-based RSM is widely used for model construction because its derivatives and integrals are easy to manipulate analytically. Denote y as the observed response variable, and $x=\left[x_{1},x_{2},\ldots ,x_{n}\right]$ as the independent variable (vector). The response variable can be approximated using regular polynomials as

(10) $$y=\beta_{0}+\sum_{i=1}^{n}\beta_{i}x_{i}+\sum_{ij\left(i\leq j\right)}^{i\in \left(1,n\right)}\beta_{ij}x_{i}x_{j}+\sum_{ijk\left(i\leq j,j\leq k\right)}^{i\in \left(1,n\right)}\beta_{ijk}x_{i}x_{j}x_{k}+\cdots +\varepsilon ,$$

where ε is the zero-mean Gaussian error variable, and $\beta =\left[\beta_{0},\beta_{1},\ldots ,\beta_{n},\beta_{11},\beta_{12},\ldots ,\beta_{nn},\beta_{111},\beta_{112},\ldots ,\beta_{nnn},\ldots \right]$ is the model parameter vector. The full RSM model with the three process parameters has a total number of 20 terms. For cases where the number of design points is less than the total number of free parameters, the analysis of variance (ANOVA) can be applied to identify the significant terms. In addition, the complicated interaction terms ($x_{i}x_{j}x_{k}$) can in most cases be omitted unless there is strong evidence supporting them. The independence of the three process parameters implies that the complicated terms can be safely omitted. For a chosen model format, the *F*-statistics can be used to verify the adequacy of the constructed surrogate model. In general, the value of F > $F_{0.05}$ indicates the model is adequate to describe the data. In this study, the final surrogate models are determined through ANOVA-based term selection under the limitation of the available design points. Therefore, only statistically significant terms are retained, and the resulting models represent reduced-order local approximations within the selected process window.

#### 4.1.1. Model for Anisotropy Indicator

The evaluation results of the quality indicators of blocks No. 1/2/3/4/5/7/8/9 given in Table 5 are used for surrogate model construction. With the aid of ANOVA, the polynomial model that describes the dependence of the anisotropy indicator $A_{\alpha }$ (unit: %) on the process parameters of torch travel speed v, arc current I, and shielding gas flow rate Q is constructed as

(11) $$A_{\alpha }=-2.171I+14.608Q+0.254vI-1.653vQ+29.681.$$

The F-value of model is 10.191, which is larger than $F_{0.05}$ = 9.117, implying the anisotropy indicator $A_{\alpha }$ is successfully captured by the surrogate models in a statistical sense. A value of $R^{2}=0.931$ also indicates the residual variance for the indicator $A_{\alpha }$ can be well explained by the independent variable in the surrogate model.

**Figure 11:**
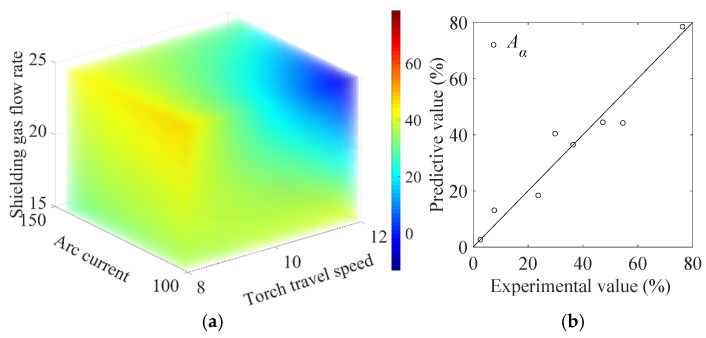
(**a**) RSM model prediction of $A_{\alpha }$, and (**b**) the comparison between the prediction and the actual experimental results.

The prediction of the anisotropy indicator $A_{\alpha }$ using Equation (11) is presented in Figure 11a, where the value of $A_{\alpha }$ is represented by the color bar. Figure 11b shows the comparison between the model prediction and the actual value of $A_{\alpha }$, where a small deviation between the two around the diagonal line is observed, demonstrating the effectiveness of the model for the purpose of prediction.

#### 4.1.2. Model for Heterogeneity Indicator

For heterogeneity modeling, two response surface models associated with the two orthogonal directions are constructed. The surrogate model for the heterogeneity indicator along the scanning path parallel to the Z-axis $CV(\alpha_{Z})$ (unit: %) is obtained as

(12) $$CV(\alpha_{Z})=7.865v-0.152I+3.418Q-0.300vQ-54.537.$$

**Figure 12:**
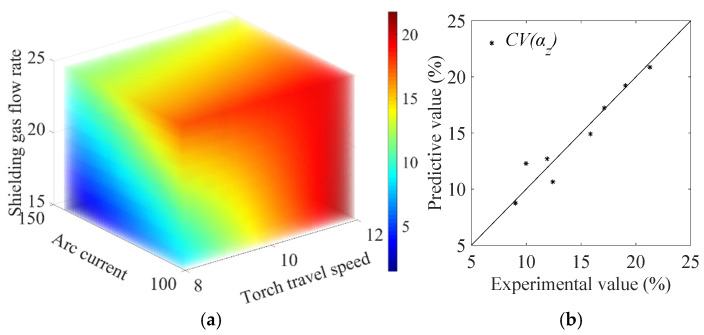
(**a**) RSM model prediction of ${CV(\alpha }_{Z})$, and (**b**) the comparison between the prediction and the actual experimental results.

The model yields an F-value of 9.229, and the adequacy of the model is statistically assured (F > $F_{0.05}$ = 9.117). The $R^{2}$ value of the resulting model is 0.925. The prediction of the heterogeneity indicator ${CV(\alpha }_{Z})$ using the model Equation (12) is made, and the results are shown in Figure 12a. The comparison between the model prediction using Equation (12) and the actual $CV(\alpha_{Z})$ from the ultrasonic testing is shown in Figure 12b.

For the heterogeneity indicator along the scanning path parallel to the Y-axis $CV(\alpha_{Y})$ (unit: %), the model is obtained as

(13) $$CV(\alpha_{Y})=-11.452v-4.795I+0.274vI-0.898vQ+0.092IQ+345.491.$$

**Figure 13:**
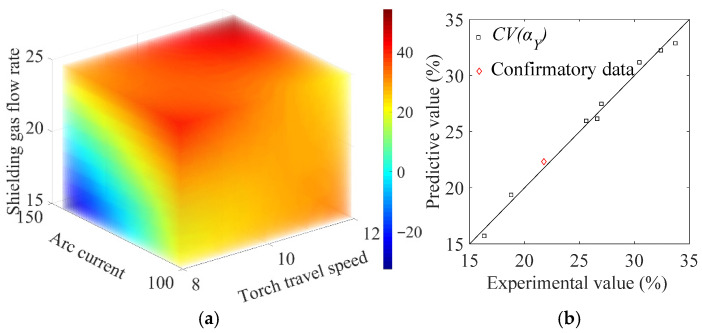
(**a**) RSM model prediction of ${CV(\alpha }_{Y})$, and (**b**) the comparison between the prediction and the actual experimental results.

The F-value of the model is 41.211 (F > $F_{0.05}$ = 28.710) with an $R^{2}$ value of 0.990, indicating that the model format is sufficient to correlate the data. The prediction of the heterogeneity indicator ${CV(\alpha }_{Y})$ using the model in Equation (13), and the comparison between the model prediction and the actual experimental results on ${CV(\alpha }_{Y})$, are shown in Figure 13a and Figure 13b, respectively. In addition, the prediction of $CV(\alpha_{Y})$ for block No. 6 in Table 5 is used to validate the accuracy of the model. The predictive value of $CV(\alpha_{Y})$ is 22.331%, as shown in Figure 13b. For the independent validation specimen No. 6, the prediction error is 2.610%, indicating that the constructed surrogate model has good predictive capability for this validation case.

The effectiveness of the constructed models in terms of statistical adequacy and prediction error is verified using ultrasonic testing data. Based on the constructed models, the process parameter optimization against different design goals is presented next.

### 4.2. Process Parameter Optimization

A general constrained optimization problem involving a real-valued function can be mathematically stated as follows:

(14) $$min\left(max\right)\;f\left(X\right),subjectto:X\;\epsilon \;\Omega ,$$

where $f\left(X\right)$ denotes the vector of the cost function, X is the vector of the independent variable, and Ω denotes the joint variable space. Optimization in this case is to search the extreme value of the objective cost function within the domain Ω.

In this study, minimizing the anisotropy $\left|A_{\alpha }\right|$ and the heterogeneity $\left|CV(\alpha_{Z})\right|$ and $\left|CV\left(\alpha_{Y}\right)\right|$ in material subjected to the valid ranges of process parameters can be formulated as the following optimization problem:

(15) $$\begin{array}{c} \min\left|A_{\alpha }\right|,|CV(\alpha_{Z})|,|CV\left(\alpha_{Y}\right)|,\\ subject\;to:\;8\leq v\leq 12,\;98\leq I\leq 146,\;15\leq Q\leq 25. \end{array}$$

In Equation (15), the terms $A_{\alpha }$, $CV(\alpha_{Z})$, and $CV\left(\alpha_{Y}\right)$ are given by Equation (11), Equation (12), and Equation (13), respectively. To obtain the optimal process parameters, the “*fmincon*” function available in the Octave package is used to solve the minimization problem. The initial value of the independent variable is set as $X_{0}=\left[8,\;98,\;15\right]$, and the lower and upper bounds are $\left[8,\;98,\;15\right]$ and $\left[12,\;146,\;25\right]$, respectively. The iterative optimization process terminates when the relative change of the objective cost function in two consecutive evaluations is less than $10^{-8}$.

#### 4.2.1. Single-Objective Optimization

**Figure 14:**
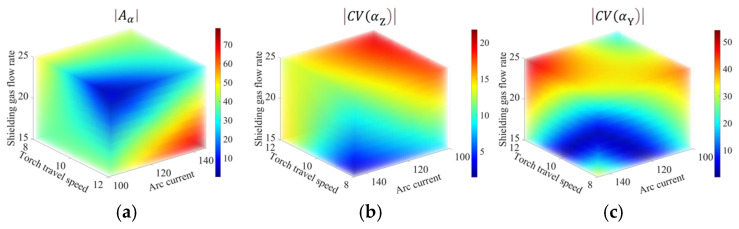
Responses of (**a**) $\left|A_{\alpha }\right|$, (**b**) $\left|{CV(\alpha }_{Z})\right|$, and (**c**) $\left|{CV(\alpha }_{Y})\right|$ in the parameter space.

For cases where a single performance is of interest, e.g., achieving directional optimal quality, the single-objective optimization can be performed for each individual indicator using the corresponding surrogate models. The optimal process parameters are obtained by solving the optimization problem of minimizing the single-objective function $\left|A_{\alpha }\right|$, $\left|CV(\alpha_{Z})\right|$ or $\left|CV(\alpha_{Y})\right|$ using the “*fmincon*” function. The results are presented in Table 6, showing that minimizing one indicator does not necessarily optimize the others. This can be visualized by the three-dimensional plots of $\left|A_{\alpha }\right|$, $\left|CV(\alpha_{Z})\right|$ and $\left|CV(\alpha_{Y})\right|$ in Figure 14a–c, respectively. For example, the minimal $\left|A_{\alpha }\right|$ and the minimal $\left|CV(\alpha_{Z})\right|$ are achieved at two distinct corners in the parameter space.

#### 4.2.2. Multi-Objective Optimization

For cases where balanced performance is sought, multiple indicators need to be considered simultaneously in the optimization operation. To achieve that, a widely used practice is to combine individual cost functions into a single one based on a certain metric [49,50]. Different optimization strategies are exemplified in this section.

(1) Overall heterogeneity indicator function

**Figure 15:**
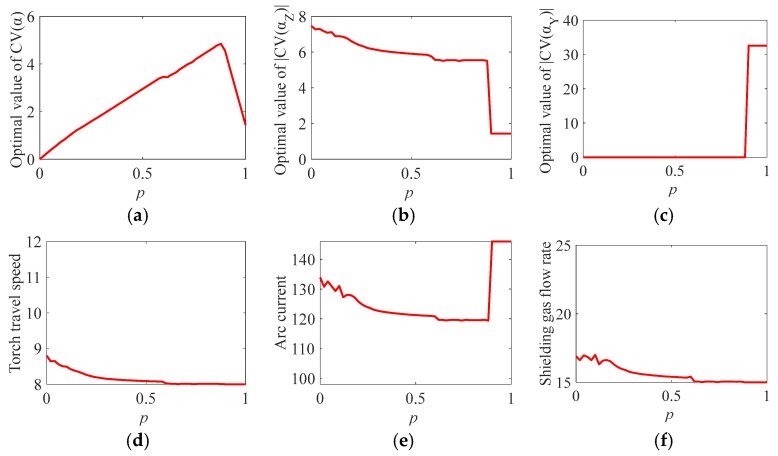
(**a**) Optimal solutions to the overall heterogeneity indicator CV(α) under different weight parameter p. The corresponding (**b**) $\left|{CV(\alpha }_{Z})\right|$, (**c**) $\left|{CV(\alpha }_{Y})\right|$, (**d**) torch travel speed value, (**e**) arc current value, and (**f**) shielding gas flow rate value.

The two heterogeneity indicators in two orthogonal directions are combined into a single heterogeneity indicator function, CV(α), to represent the overall heterogeneity for parameter optimization. Since the optimization aims to achieve balanced heterogeneity control in both directions, the weight coefficient p is introduced to account for relative contributions to the overall objective. In this case, the overall heterogeneity function can be expressed as

(16) $$CV\left(\alpha \right)=p\cdot \left|CV\left(\alpha_{Z}\right)\right|+\left(1-p\right)\cdot \left|CV\left(\alpha_{Y}\right)\right|,$$

where $p\in \left[0,\;1\right]$. With Equation (16), solutions to the minimal overall heterogeneity CV(α) under different weight parameter p are obtained and are shown in Figure 15a. The corresponding values of the individual indicators $\left|{CV(\alpha }_{Z})\right|\;$and $\left|CV(\alpha_{Y})\right|$ are shown in Figure 15b and Figure 15c, respectively. The optimal values of the three process parameters are presented in Figure 15d–f.

The optimization results reveal that the optimal torch travel speed v and shielding gas flow rate Q are less than 9 mm/s and 18 L/min, respectively, for an arbitrary p. The result implies that the shielding gas flow rate of 18 L/min is sufficient to protect the arc from air pollution. It provides quantitative support on using the low flow rate to avoid introducing potential spatters in the molten pool during the WAAM process. The process parameters from the optimal solutions shown in Figure 15 at three arbitrarily chosen values of p are presented in Table 7 for the purpose of illustration.

(2) Overall anisotropy and heterogeneity indicator function

The composite desirability function (CDF) [49] is adopted for optimizing multiple responses with possible conflicting nature. The CDF can be expressed as

(17) $$CDF={\left(\prod_{i=1}^{n}{d_{i}(y)}^{w_{i}}\right)}^{1/\sum_{i=1}^{n}w_{i}},$$

where the term $w_{i}$ is the weight for the ith response, which assigns the importance of the response relative to other responses, $d_{i}(y)$ is the individual desirability defined for the ith targeted response, and y is the response value. For smaller-the-better (STB) type controllable response, the desirability function $d_{i}(y)$ can be set as follows:

(18) $$d_{i}\left(y\right)=\left\{\begin{array}{c} 1,\;y<T\\ \frac{U-y}{U-T},\;T\leq y\leq U\\ 0,\;y>U \end{array},\;\right.$$

where the term U is the upper limit of a response, and T is the target value of the individual response. The value of individual desirability $d_{i}\left(y\right)$ or composite desirability CDF varies between 0 and 1. A unity value represents an ideal situation and zero means that one or more of the responses are not within the desired acceptable limits. The values of the weight can be determined based on engineering experience.

For demonstration, Table 8 shows the response variables, the example target, upper limit, and weight. It is considered that the overall material quality is undesirable once the value of anisotropy or heterogeneity indicator function exceeds 50%. The weighting coefficients $q_{1}$ and $q_{2}\;$are varied from 1 to 5 to evaluate the influence of different desirability sensitivities on the compromise solution between the two response criteria.

Based on the anisotropy indicator model, Equation (11), and the overall heterogeneity indicator function, Equation (16), the CDF of overall anisotropy and heterogeneity indicator is assumed as

(19) $${CDF}_{ACV}={d_{\left|A_{\alpha }\right|}}^{q_{1}}\cdot {d_{\left|CV\left(\alpha \right)\right|}}^{q_{2}}.$$

**Figure 16:**
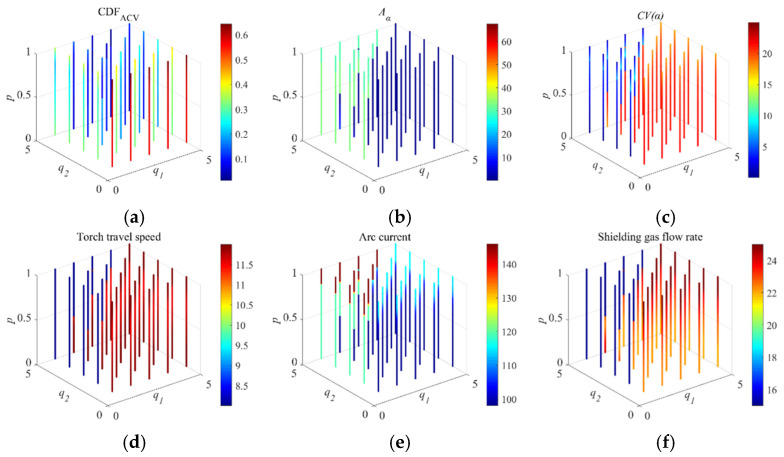
Optimal solutions obtained by optimizing ${CDF}_{ACV}$ with different weights “$q_{1},q_{2},p$”. (**a**) ${CDF}_{ACV}$, and (**b**–**f**) are the resulting $\left|A_{\alpha }\right|$, CV(α), torch travel speed value, arc current value, and shielding gas flow rate value, respectively.

In this way, the two types of indicators are combined into a single objective function (${CDF}_{ACV}$), which can subsequently be resolved using the “*fmincon*” function with ($-{CDF}_{ACV}$) as the cost function, as described before. The ${CDF}_{ACV}$ results, the corresponding responses, and optimal process parameters under different weights $q_{1}$, $q_{2}$, and p are obtained, and are shown in Figure 16, where their values are indexed by the color bar.

In practical engineering applications, once the relative importance of the quality indicators is specified, the corresponding optimal solution can be determined using the proposed composite desirability framework. Table 9 summarizes the optimal $CDF_{ACV}$ values, the corresponding response values, and the associated process parameters for representative combinations of $q_{1}$, $q_{2}$, and p. The results show that the optimal solution is sensitive to the selected weights. Increasing the weight assigned to $|A_{\alpha }|$ tends to reduce the anisotropy indicator, whereas increasing the weight assigned to ∣CV(α)∣ favors a lower heterogeneity indicator. This indicates that the two partial responses cannot generally reach individual optima simultaneously and that a compromise solution is required. In addition, several optimum responses are located near the boundary of the investigated parameter space, suggesting that the best trade-off within the current design range is often achieved at the edge of the selected process window. Combined with the microstructural observations, these results further indicate that the selected process parameters affect the final quality through influence on pore distribution and grain morphology.

## 5. Conclusions

This study developed a systematic approach to WAAM process parameter optimization. The minimizations of the anisotropy and heterogeneity in the microstructure of the material along two orthogonal principal directions were sought. A total of nine WAAM blocks were fabricated using different combinations of the three major process parameters. An ultrasonic method was developed allowing for volumetric evaluations of anisotropy and heterogeneity, using velocity and attenuation coefficient as indicators. Metallographic images reveal that the non-uniform distribution of pores and the variation of microstructural features collectively contribute to the anisotropy and heterogeneity. Surrogate models based on the response surface method are established to correlate the ultrasonic indicators with three WAAM process parameters. Single- and multi-objective optimizations were demonstrated. Based on the current results, the following conclusions are drawn.

(1) Ultrasonic velocity and attenuation provide complementary indicators for characterizing anisotropy and heterogeneity. Velocity-based anisotropy remains below 0.53%, indicating nearly isotropic elastic stiffness along the two principal directions.

(2) Attenuation-based anisotropy reaches values as high as 76%, revealing pronounced direction-dependent microstructural and porosity features. Metallographic analysis confirms that interlayer pore enrichment and grain morphology variation jointly govern the attenuation response.

(3) The attenuation coefficient is significantly more sensitive to microstructural heterogeneity than velocity, demonstrating its suitability as a volumetric, non-destructive indicator for WAAM quality assessment.

(4) Response surface surrogate models successfully correlate ultrasonic indicators with process parameters, enabling both single- and multi-objective optimization within the feasible process window. The proposed approach provides a systematic alternative to conventional trial-and-error parameter tuning.

Overall, the results indicate that WAAM 2319 aluminum alloy fabricated within the investigated process window exhibits nearly isotropic elastic stiffness, while still retaining notable microstructural heterogeneity along the build height. The novelty of this study lies in integrating ultrasonic characterization, microstructural observation, and multi-objective optimization into a unified framework for WAAM quality evaluation and process parameter optimization. Although the individual methods employed are well established, their combined application in the present study provides both a practical strategy for microstructure-informed optimization and a scientific basis for elucidating the process–structure–quality relationships in wire and arc additive manufacturing.

## Figures and Tables

**Table 1:** Chemical composition of ER2319 aluminum wire (wt.%).

	Si	Fe	Cu	Mn	Mg	Zn	Ti	Cr	Be	Zr	V	Al
ER2319	0.106	0.156	5.95	0.273	0.0086	0.012	0.104	0.0031	0.0001	0.104	0.068	Rest

**Table 2:** The key process parameters of WAAM.

Label	Process Parameter	Unit	Level 1	Level 2	Level 3
v	Torch travel speed	mm/s	8	10	12
I	Arc current	A	100	120	140
Q	Shielding gas flow rate	L/min	15	20	25

**Table 3:** The actual process parameters are associated with the WAAM blocks.

No.	v (mm/s)	I (A)	Q (L/min)
1	8	98	15
2	8	119	20
3	8	139	25
4	10	101	25
5	10	119	15
6	10	139	20
7	12	101	20
8	12	119	25
9	12	146	15

**Table 4:** The velocity-based anisotropy and heterogeneity results.

No.	${\bar{V}}_{Z}$ (m/s)	${\bar{V}}_{Y}$ (m/s)	$A_{V}$ (%)	*CV*(*V*_Z_) (%)	*CV*(*V_Y_*) (%)
1	6235.608	6221.461	0.227	0.395	0.222
2	6194.151	6189.810	0.070	0.136	0.183
3	6214.694	6195.316	0.312	0.164	0.325
4	6207.806	6222.981	0.244	0.156	0.180
5	6236.099	6203.600	0.523	0.194	0.229
6	--	6126.451	--	--	0.243
7	6205.447	6199.178	0.101	0.045	0.373
8	6198.682	6167.777	0.500	0.040	0.820
9	6202.933	6191.922	0.178	0.038	0.277

**Table 5:** The attenuation-based anisotropy and heterogeneity results.

No.	${\bar{\alpha }}_{Z}$ (Np/m)	${\bar{\alpha }}_{Y}$ (Np/m)	$A_{\alpha }$ (%)	CV(*α*_Z_) (%)	CV(*α*_Y_) (%)
1	8.246	5.707	36.394	9.015	26.619
2	6.128	4.536	29.857	12.422	18.788
3	6.471	3.999	47.221	11.904	32.409
4	3.930	4.985	23.668	19.044	30.447
5	7.822	4.470	54.540	9.981	16.338
6	--	4.7583	--	--	21.765
7	3.950	3.658	7.676	21.277	25.619
8	5.133	5.000	2.625	17.112	33.732
9	6.326	2.832	76.305	15.857	27.008

**Table 6:** Optimal process parameters obtained by single-objective optimization.

Objective	v (mm/s)	I (A)	Q (L/min)	Optimal Response (%)	Responses of Other Indicators (%)
min $\left|A_{\alpha }\right|$	11.576	103.611	24.035	$\left|A_{\alpha }\right|$ = 1.984 × 10^−7^	$\left|{CV(\alpha }_{Z})\right|=19.464;\;\left|{CV(\alpha }_{Y})\right|=23.862$
min $\left|CV(\alpha_{Z})\right|$	8.000	146.000	15.000	$\left|{CV(\alpha }_{Z})\right|\;=$ 1.438	$\left|A_{\alpha }\right|=29.658;\;\left|{CV(\alpha }_{Y})\right|=32.589$
min $\left|CV(\alpha_{Y})\right|$	8.798	133.894	16.900	$\left|{CV(\alpha }_{Y})\right|\;=$ 2.942 × 10^−9^	$\left|A_{\alpha }\right|=38.795;\left|CV(\alpha_{Z})\right|=7.452$

**Table 7:** Optimal solutions obtained by overall heterogeneity indicator optimization.

Objective	p	CV(*α*) (%)	Individual Indicators (%)	v (mm/s)	I (A)	Q (L/min)
min CV(α)	0.5	2.952	$\left|{CV(\alpha }_{Z})\right|$ = 5.905$;\;\left|{CV(\alpha }_{Y})\right|$ = 3.888 × 10^−9^	8.008	121.253	15.403
	0.8	4.435	$\left|{CV(\alpha }_{Z})\right|$ = 5.544$;\;\left|{CV(\alpha }_{Y})\right|$ = 1.177 × 10^−7^	8.010	119.582	15.046
	0.9	4.553	$\left|{CV(\alpha }_{Z})\right|$ = 1.437$;\;\left|{CV(\alpha }_{Y})\right|$ = 32.589	8.000	146.000	15.000

**Table 8:** The criteria for establishing composite desirability function.

Response	Target T	Upper U	Weight
$\left|A_{\alpha }\right|$	0	50%	$q_{1}=1,2,3,4,5$
|CV(α)|	0	50%	$q_{2}=1,2,3,4,5$

**Table 9:** Optimal solutions obtained by overall anisotropy and heterogeneity indicator optimization.

Objective	$q_{1}$	$q_{2}$	p	v (mm/s)	I (A)	Q (L/min)	Optimal Response (%)	Responses of Individual Indicator (%)
max (${CDF}_{ACV}$)	1	1	0.5	11.645	98.779	23.019	0.577	$\left|A_{\alpha }\right|$ = 6.263 × 10^−7^; $\left|CV\left(\alpha \right)\right|$ = 21.151
	5	5		11.653	98.019	22.869	0.064	$\left|A_{\alpha }\right|$ = 1.461 × 10^−7^; $\left|CV\left(\alpha \right)\right|$ = 21.110
	1	5		8.010	119.583	15.048	0.248	$\left|A_{\alpha }\right|$ = 33.530; $\left|CV\left(\alpha \right)\right|$ = 2.772
	5	1		11.664	98.199	22.870	0.578	$\left|A_{\alpha }\right|$ = 0.578; $\left|CV\left(\alpha \right)\right|$ = 3.205e-7
	1	1	0.8	11.718	109.159	24.564	0.596	$\left|A_{\alpha }\right|$ = 1.184 × 10^−7^; $\left|CV\left(\alpha \right)\right|$ = 20.214
	5	5		11.741	111.591	24.911	0.075	$\left|A_{\alpha }\right|$ = 9.206 × 10^−7^; $\left|CV\left(\alpha \right)\right|$ = 20.195
	1	5		8.002	119.411	15.009	0.209	$\left|A_{\alpha }\right|$ = 33.458; $\left|CV\left(\alpha \right)\right|$ = 4.403
	5	1		11.780	109.923	24.527	0.596	$\left|A_{\alpha }\right|$ = 4.917 × 10^−7^; $\left|CV\left(\alpha \right)\right|$ = 20.213
	1	1	0.9	11.936	114.508	24.907	0.619	$\left|A_{\alpha }\right|$ = 4.486 × 10^−8^; $\left|CV\left(\alpha \right)\right|$ = 19.061
	5	5		11.999	115.935	24.999	0.092	$\left|A_{\alpha }\right|$ = 1.946 × 10^−7^; $\left|CV\left(\alpha \right)\right|$ = 18.968
	1	5		8.000	145.999	15.000	0.252	$\left|A_{\alpha }\right|$ = 29.658; $\left|CV\left(\alpha \right)\right|$ = 4.553
	5	1		11.931	114.436	24.907	0.619	$\left|A_{\alpha }\right|$ = 2.249 × 10^−7^; $\left|CV\left(\alpha \right)\right|$ = 19.065

## Data Availability

The original contributions presented in this study are included in the article. Further inquiries can be directed to the corresponding author.
